# The Duality of the Mind Proved by the Structure, Functions, and Diseases of the Brain, and by the Phenomena of Mental Derangement, and Shown to Be Essential to Moral Responsibility

**Published:** 1845-07

**Authors:** 


BRITISH AND FOREIGN

### MEDICAL REVIEW,

FOR JULY, 1845.

### PART FIRST.

### Analytical and Critical Reviews.

### Art. I.

*The Duality of the Mind proved by the Structure, Functions, and Diseases of the Brain, and by the phenomena of Mental Derangement, and shown to be essential to Moral Responsibility.* By A. L. Wigan, M.D.—*London,* 1844. 8vo, pp. 460.

"In looking over the following pages for the purpose of making an index to their contents, I am struck with the conviction, that (were it now to be commenced,) it would be easy to execute the task much better." These are the first lines of the author's preface; and we venture to say that there are none truer in the book. For of all the loose, ill-digested treatises, that have ever come under our critical notice, we think we may fairly say that none have surpassed this. The book, however, will be found pleasant reading enough, for those who think more of liveliness of style, and pointedness of expression, and well-told pretty stories, (with which it is plentifully scattered,) than of understanding the author's aim, and keeping hold of the thread of his argument. But we doubt if any such reader would be able to give a much better account of the book than its title-page sets forth; indeed we question whether he would not be so bewildered, as to have lost even what he had learned from it. And yet, even considered as a scientific production, it has its merits. It is eminently suggestive. It puts many old facts in a new light. It attempts (to say the least) to solve certain difficulties in the explanation of mental phenomena, which have baffled the ablest thinkers. And the author is most firmly and unmistakeably convinced of the truth of his positions, and puts them forth with an earnestness and sincerity, which cannot but inspire respect. There is, we think, no *sham* about him; which, in these days of pretension, is saying a great deal. We are not quite so sure, however, as to the genuineness of some of his narratives; in some of which the worthy Doctor seems to have had his evident good-nature a little practised upon; whilst in others, for which he personally vouches, we question whether a certain liveliness of imagination has not sometimes relieved the bare truth with a little embroidery. This suspicion may seem inconsistent with what we have just predicated, touching our estimate of our author's character. But it is not so in reality; since we are very sure that he thoroughly believes everything that he has written. This conviction, moreover, he enforces so frequently, telling us so often of the long growth and deep root of his opinions, in his mind, that it has much of the appearance of dogmatism. Dr. Wigan *knows* his opinions to be *truth;* and so they are according to the etymology of the word, being "that which he troweth." But because they are *his* truth, there is no reason why they should be *ours*, or *our readers'*, without examination; since certain arguments may arise in our minds, certain facts may have presented themselves to our observation, which may possibly lead us to a different view. This possibility does not seem to have entered into the amiable writer's mind; for having (as he frequently assures us,) conceived his opinions thirty years ago, and given them the full maturity of that lengthened gestation, he seems to have fallen in love with them, (like Prometheus with his animated statue,) and to have allowed them to become so ingrafted with his own entity, that Dr. Wigan and Duality, Duality and Dr. Wigan, will henceforth become inseparable. Assuredly *we* shall not attempt to dissolve this union; satisfied that the two must be coexistent, as long as Dr. Wigan remains in this mortal sphere; and when the union is dissolved, we doubt if the "Duality," in its present form at least, will survive the loss of its affectionate parent.

Now we have, for our readers' sake, endeavoured to seek out the purpose of this book, and to put it into a form in which it may be presented to them; because, as we just now said, it *has* merit, and should not be overlooked by those, who direct their reasoning or their speculations into that dark and intricate track, which connects the domains belonging severally to mind and body. Our sketch of its aim and purport must be anything but an analysis of the book as it stands; since this would be more unintelligible than the work itself; but is intended to represent what, in the phrase of the poets, is the *argument*, whose development is the author's intention. Our labour is in some degree lightened by the summary of propositions proved, or intended to be proved, by the author, which is given in his Fourth Chapter; but it has been in finding the proofs, or what Dr. Wigan conceives to be the proofs, of these, that our labour has been chiefly exercised; and if we have in any way done him injustice, by misrepresenting or insufficiently stating them, we can only regret that we should have been misled by the difficulties to which we have alluded, as arising out of Dr. Wigan's own mode of putting them forward.

Not to weary the patience of our readers, we shall not present them with the whole of Dr. Wigan's twenty propositions at one quotation; but shall first extract and discuss those, which may be regarded as fundamental; postponing for the present those which are erected upon the preceding, and which must fall if *they* are proved to be foundationless. For this purpose, we must slightly change the order in which they appear that all those relating to each division of the subject may be placed together.

"I believe myself," says Dr. Wigan, "able to prove :—

1. "That each cerebrum is a distinct and perfect whole, as an organ of thought.

2. "That a separate and distinct process of thinking or ratiocination may be carried on in each cerebrum simultaneously.

3. "That each cerebrum is capable of a distinct and separate volition, and that these are very often opposing volitions.

4. "That, in the healthy brain, one of the cerebra is almost always superior in power to the other, and capable of exercising control over the volitions of its fellow, and of preventing them from passing into acts, or from being manifested to others.

5. "(14.) That one cerebrum may be entirely destroyed by disease, cancer, softening, atrophy, or absorption; may be *annihilated*, and in its place a yawning chasm; yet the mind remain complete and capable of exercising its functions, in the same manner and to the same extent, that one eye is capable of exercising the faculty of vision when its fellow is injured or destroyed; although there are some exercises of the brain, as of the eye, which are better performed with two organs than one. In the case of vision, the power of measuring distances for example; and in the case of the brain, the power of concentrating the thoughts upon one subject, deep consideration, hard study; but in the latter case it is difficult to decide how far the diminished power depends on diminution of general vigour from formidable and necessarily fatal disease.

6. "(15.) That a lesion or injury of both cerebra is incompatible with such an exercise of the intellectual functions, as the common sense of mankind would designate *sound mind*.

7. "(16.) That from the apparent division of each cerebrum into three lobes, it is a natural and reasonable presumption, that the three portions have distinct offices, and highly probable that the three great divisions of mental functions laid down by phrenologists, are founded in nature; whether these distinctions correspond with the natural divisions is a different question; but the fact of different portions of the brain executing different functions, is too well-established to admit of denial from any physiologist.

8. "(17.) That it is an error to suppose the two sides of the cranium to be always alike; that, on the contrary, it is rarely found that the two halves of the exterior surface exactly correspond; that indeed, in the insane there is often a notable difference, still more frequent in idiots, and especially in congenital idiots.

9. "(20.) That every man is, in his own person, conscious of two volitions, and very often conflicting volitions, quite distinct from the government of the passions by the intellect; a consciousness so universal, that it enters into all figurative language on the moral feelings and sentiments, has been enlisted into the service of every religion, and forms the basis of some of them, as the Manichæan." (pp. 26-30.)

Dr. Wigan's mode of demonstrating these propositions is somewhat original. He begins by demanding our temporary assent to them, and promises the evidence hereafter. If that evidence is found to be defective, he tells us to discard and deny his dogmas. If on the other hand, he adduces apparently conclusive evidence in their behalf, we presume (though he does not say so) that he expects us to retain them. Now by this mode we will undertake to prove almost *any* proposition; since it would be difficult to invent one so absurd, that there could be nothing said in its favour. First, (this is Dr. Wigan's recipe,) catch a proposition. Next, assume it to be true. Then bring forward the arguments in its favour. If these fit, your proposition may stand. If they don't, (and he must be but a poor advocate who cannot make them,) your proposition must fall. But so long as, having been once set upon its feet, the idol can keep its balance, (all rude assaults of opposing arguments having been carefully kept out of the way,) so long you are to bow down and worship it. By the English law, every man is presumed to be innocent, until he is proved to be guilty. In Dr. Wigan's code of logic, everything is true, which has not been proved to be false. Now we shall endeavour to supply the deficiency, by showing that, whatever may be the amount of *probability* to which Dr. Wigan's speculations may lay claim, the very nature of the subject forbids our regarding any of them as *proved,* or even as furnishing a solid foundation on which to raise further argument. What, for example, can be more inconclusive than the following argument from analogy, which we find at the outset?

"To me it seems that the provision of two distinct and perfect brains for this object is like the provision of two eyes and two ears. In thought, as in vision and in hearing, each organ may suffice to perform perfectly all its appropriate functions, yet the two when in health produce only one result. We have only one sound with both ears, each of them hearing it at the same time. We see only one object with both eyes, each seeing it separately at the same time. We carry on only one train of thought in both brains; each thinking it at the same time; all this, however, is contingent, not only on the perfect health of the organs, but on their due exercise and cultivation. In disorder or disease, brain, eye, and ear, convey separate, distinct, conflicting ideas,—one, or both, necessarily erroneous." (p. 35.)

Does Dr. Wigan seriously mean that it is the eye which sees, and the ear that hears, and that the *sensorium* has nothing to do with the process? Is he ignorant that what we see is *not* the image formed upon *either* of our two retina, but that the mind, receiving both, blends them by an unconscious act into a representation of the object, which (if this be not very remote,) neither eye could alone communicate? Would not the connexion of one of our eyes with another sensorium, if such a thing can be deemed possible, give rise to an impression in *that* sensorium, thus arousing a second consciousness? And is not the very *oneness* of the representation we derive from the blending of the two images which are pictured on the retinæ, a powerful argument for the unity, and *against* the duality of the mind? To us it seems that the first part of this argument of Dr. Wigan's entirely fails; and that all the information furnished to us by our senses tells of a mind "one and indivisible." Nor does the second part strike us as more successful. For if one eye is so injured or defective, as to convey sensations which are altogether different from those of the other, the case is precisely the same as when the two eyes are directed to different objects, (which may be easily accomplished,) or when the two ears have different sounds conveyed to them. For the *one* mind is then conscious of *two* distinct sensations, and will be perplexed by them if they are discordant, unless the attention be fixed upon either of them, separately, which the mind has the power of doing, in the same manner as it can attend to any one out of *a large number* of distinct sensations, conveyed by the several organs of sense. We shall hereafter see that this state *of attention* is regarded by Dr. Wigan as attributable to the consentaneous action of both hemispheres; but he does not explain to us how the attention of either hemisphere can be fixed upon the impressions conveyed by the nerves of the opposite side, since, according to him, the *corpus callosum,* instead of being a commissure or bond of union between the two hemispheres, as commonly regarded, is a wall of separation between them. (p. 16.) He distinctly states that "the fibres from each half of the brain only go to the middle line, and do not pass over to the other side;" a somewhat dogmatic assertion for a man who prefers no claims to anatomical authority. But he admits in his preface that his assertions respecting the structure and office of the corpus callosum require modification; leaving us much in the dark, however, to what extent, or in what manner, this is to be understood; and only intimating that his views as to the nature of the mind are not thereby affected.

The most important evidence adduced by Dr. Wigan in support of his views, is that derived from cases in which one of the hemispheres has been destroyed by disease or injury; and in which neither loss of intellectual power, nor any functional disturbance, has been apparent. Of well-authenticated cases of this kind, there is certainly no want; and Dr. Wigan is quite justified in stating that one such case is quite as conclusive as a hundred, in proving that each hemisphere, (or, as he prefers to call it, each brain,) is a perfect instrument of thought and ratiocination. But the proof is wanting that a separate mind, that is to say *a distinct consciousness,* can ever at once be present in each. We may be misrepresenting Dr. Wigan in saying that he affirms this; but if we do, it is his own fault. Two *consentaneous volitions,* in our apprehension, involve two *distinct* centres of power, two *egos* in one person; and two *egos* must have two *consciousnesses,* as distinct as those of the Siamese twins. We do not think that there is any evidence for the allocation of the *consciousness,*—which we take to be the essence of mind,—in the cerebral hemispheres. The study of comparative anatomy, physiological experiment, and pathological investigation, seem to lead irresistibly to the conclusion, that these organs are but the instruments of but *one class* of mental phenomena, and that they are *not* the peculiar seat of consciousness; in other words, that the sensorium is *not* in them. Hence to found any argument as to the duality of the *mind* upon the repetition of parts in the two *hemispheres,* and the power of either of them to act alone, seems to us utterly illogical. The mind *may,* and probably *does,* possess the power of using *both* as instruments of intellectual operations, to arrive at *one* result, in the same manner as it uses both eyes to look at one object; and there seems to us no more reason to suppose that, in any ordinary acts of mind, only one cerebrum is acting, and that for *attention* both are required, than there is to suppose that only one eye is employed at a time in ordinary vision. Even discrepant actions of the two hemispheres may, for anything we know to the contrary, be harmonized and blended in the central seat of mind; in precisely the same manner that the two flat pictures formed in the retinæ are fused, as it were, together, so as to form the visual image of a solid. All analogy seems to us in favour of this view. We do not assume it as *proved;* but it is at least as *probable* as Dr. Wigan's; and harmonizes better with what we know of mental phenomena. It is possible that Dr. Wigan may say that there is no essential difference between his view and ours. If there be not, why then we cannot see the novelty of Dr. Wigan's; since ours is nothing different from that which physiologists have been long accustomed to maintain, only, perhaps, somewhat more precise in its form.

Dr. Wigan has not referred to the evidence of comparative anatomy as either supporting or opposing his views. To us it seems that the facts which it supplies afford him no support; and that some of them rather militate against his doctrines. In every instance in which there is a lateral doubling of the nervous centres, there is a well-marked *commissural* band, which evidently has for its office to connect the operations of the two sides. The passage of fibres from one lateral half to the other, can be readily and distinctly traced; and we see no reason whatever, from the actions of the lower animals, to imagine that two opposing trains of thought are ever going on at once in their minds, or that two volitions are struggling for mastery over the body. Now in the radiata, the ganglia which stud the nervous circle appear to be all equal in their endowments. Thus in the star-fish, the whole body being constructed upon a perfectly symmetrical plan, every one of the radii resembles the rest; the mouth is in the centre, and all the organs of sensation and motion are uniformly disposed around it. Hence we cannot regard any of the ganglia, (which are never less than five in number,) as having a presiding function over the rest, or as being the peculiar seat of sensation; and there is evidence that one or more of them may be removed, without any great discomposure of the general functions of the body. Hence, on Dr. Wigan's hypothesis of duality as applied to man, the mind (such as it is) of the five-rayed star-fish must be *quinary;* and that of the twelve-armed species must be *duodecimal.* It may be difficult to prove that this is *not* the case; but it would seem to us still more difficult to prove that it *is;* and in default of that proof, the matter must rest as it was before Dr. Wigan took it up.

After all the pains we have taken to arrive at Dr. Wigan's exact opinions as to the amount or degree of distinctness between the operations of the two halves of the brain, we can find nothing clearer than the following passage.

"One of two things must be: either each hemisphere or cerebrum is a perfect whole, capable of exercising all the functions which, in the aggregate, form *the mind* of the individual; or else each half must exercise some of these functions, and the other half the remainder, so as between them to make up *a* mind. There is positively no other thing possible, the mind is performed completely by each brain, or jointly by the two." (p. 47.)

Now it seems to us that, if the first proposition be true, (as Dr. Wigan asserts,) there are in reality *two minds;* and that the alternative is not confined to the second. For if, as we have suggested, the essential seat of mind be not in the cerebral hemispheres at all (and the absence of any representative of these hemispheres in the greater number of invertebrata seems decisive in favour of such an idea,) and if those organs be only employed as its instruments in a certain class of operations, it seems quite legitimate to admit that they may usually work together as a whole, whilst either is capable of operating alone. Two analogous examples,—the one drawn from the inorganic, the other from the organic world,—may render our meaning more apparent. If a bar magnet,—which has, of course, the north pole at one end, and the south pole at the other,—be cut or broken across the middle, each half becomes a perfect magnet; a new north and another south pole manifesting themselves at the points of separation. Yet the first magnet was not made up of the two which were formed by the division; it was *one,*—capable, however, of being separated not only into two, but into any number of minor magnets, each complete in itself. Again, most of our readers are probably familiar with the circulation of fluid, which takes place in the chara, within the long cells that form its stem and branches. The fluid passes along one side of each cell, from end to end; then returns along the opposite side; and then again follows its first track. Now if a ligature be put round the middle of the cell, so as to divide it into two, this will not check the circulatory action; on the contrary, it will establish two currents, one in each cell; and these will go on as regularly as if the interruption had not been made. Here, then, are two instances in which we can divide *a whole* into parts, of which each shall perform its functions; and yet the functions of the whole were neither the mere aggregate of those of the parts, nor had the functions of the parts an independent existence, until the division was made. Thus even if Dr. Wigan could prove that, by slicing the nervous centres down the median line, so as to isolate them completely, he could make two sentient beings out of one, he would not prove that two distinct centres of mental action *previously* existed in the individual.

We have now considered the entire anatomical argument adduced by Dr. Wigan in proof of his fundamental position, that "each cerebrum is a distinct and perfect whole as an organ of thought;" and have endeavoured to show that it affords no sufficient ground for this assumption,—because, in the first place, there is no proof of the localization of *consciousness,* the mainspring (as it were) of all mental operations, in the cerebrum at all, but very much that indicates the contrary; and because in the next place, the complete duality of function is not required by the fact of the repetition of the same parts in both, nor by the circumstance that when one hemisphere is in abeyance, the remaining one can execute the operations of the entire organ. We next turn to the metaphysical part of the question; and shall inquire how far Dr. Wigan is justified in his twentieth proposition, (quoted by us in page 3, as the 9th,) that every man is occasionally conscious of two volitions, very frequently of a conflicting nature, quite distinct from the government of the passions by the intellect.

This assertion we cannot meet in any other way than by a simple denial. We have long been in the habit of analysing our own mental operations, and of endeavouring to determine their sources; and we have never felt the necessity of having recourse to such an idea as that of two *volitions,* which (as we have already remarked) seems equivalent to that of two *egos.* It appears to us that all human actions (excluding the reflex) may be regarded as either *automatic* or *voluntary.* The former group includes those which are separately classed as *consensual, instinctive,* and *emotional,* together with some which were once voluntary, but which have become *habitual.* The will may generally restrain these actions; but if the will be weak, or the stimulus which prompts them be strong, they may be performed in spite of it. On the other hand, all *voluntary* actions are the result of intellectual processes, which end in a *determination* that operates upon the motor nerves. The nature of this determination depends entirely upon the *motives* which have been in operation; for we believe it to be an established axiom amongst metaphysicians, that any two sane minds *must* arrive at the same conclusions, provided the motives are the same. Now it is very well known to every one, that these motives may be numerous and conflicting; in fact there is no limit to their possible number, and to the variety of their direction. Sometimes they act in *direct* antagonism to one another, and leave no middle course; thus the Acquisitive propensity may produce a desire to commit a theft, whilst the Conscientiousness acts to restrain it.

The determination or *will*, resulting from the operation of these conflicting motives, will be *for* or *against* the action, according as the acquisitiveness or the conscientiousness be stronger. The conscientiousness being unduly weak, or the acquisitiveness being unduly strong, the determination will be in favour of the action; which the *will*, acting upon the motor organs, then puts into operation. Of course, if the conscientiousness be victorious the result will be the contrary. We have here supposed only the simplest possible case; that in which two opposing propensities only are in operation. But the motives are seldom in reality so easily analysed. Other propensities may be in action; and even the same propensity may suggest two opposing motives. Thus Benevolence may suggest to the possible thief that the individual whom he is about to rob will be injured thereby; whilst Selfishness will lead to the consideration of the benefits he will himself derive from the appropriation; or even Benevolence itself may urge to the theft, by representing the good which may be done to others with the property. Again, the motives may be themselves more purely the result of reasoning processes; as for instance, when the chances of detection and punishment, or of escape and immunity, are weighed against each other. Here, then, are a large number of *pros* and *cons*, the estimate of which will vary according to the nature of the circumstances, and the habitual strength of different propensities. Two individuals, in precisely the same circumstances may arrive at an opposite conclusion; because the very same conditions will be presented to their intellect as motives of different strength, according to the relative development of the different propensities which they call into action—these last, not the circumstances themselves, being the real weights in the scale. For in appreciating the value to be attached even to the results of reasoning,—as upon such a subject as the probability of detection and punishment,—it is the degree of the moral feeling *fear*, that will govern the weight of the motive. The hitherto virtuous youth, placed in circumstances of temptation, knowing that detection must blast his character and be fatal to his prospects in life, will experience this motive with much greater force, than the practised thief, who has only to look for a few months of confinement and hard labour, a slight and not always unpleasing variation from his ordinary course, as the consequence of his misdoing.

Now in the case we have supposed, there is no middle course. "To *do*, or not to *do*, that is the question." How many a fierce conflict has been sustained, of which the world knows not, between the two opposing classes of motives, in minds which are commonly regarded as neither exposed, nor likely to yield, temporarily to such temptations! How many a time has the will given itself up to the sway of the evil propensity, the hand been stretched forth to grasp the tempting object; when some new motive, or the sudden upstart of one that had been already repressed, some pious precept, or some lingering memory of affection, has come in time to change the course of the will, and to save from the commission of crime, drawing back the hand, and causing flight from the scene of temptation! We remember to have somewhere read the story of a poor barber, who was summoned to perform his office upon a wealthy man, upon whose table lay a large amount of gold. After going through the preliminaries, and applying his razor to the chin of his subject, the operator precipitately fled, leaving his work unfinished. On being summoned, and interrogated as to the cause of his strange conduct, he stated that the sight of the glittering coin, the thought of his starving family, the facility with which he might possess himself of the money by the murder of the gentleman, altogether tempted him so strongly, that he could not have restrained himself from the commission of the crime, if he had continued in the room; and he therefore obeyed his good impulses, by seeking safety in flight.

Now in all such cases as this, Dr. Wigan would have us believe, that the two brains (as he calls them) are at war with one another, and that the strongest gets the victory. Of this, there seems to us an utter absence of proof; and the hypothesis is attended with numerous difficulties. For instance, we must suppose that the *pros* are all mustered on one side of the head, and the *cons* on the other. What should determine this arrangement, we must confess ourselves unable to see. In any case in which one hemisphere is functionally in abeyance, no such conflict as this could, on Dr. Wigan's principles, take place; and either the propensity would act unrestrainedly, or it would not act at all. The hypothesis is completely unnecessary; because if it be once conceded, (and this Dr. Wigan does,) that the different propensities have their seat in different parts of the brain, and that all the organs are present in each hemisphere, the conflicting actions we have alluded to may take place just as well in one hemisphere, as in two conjoined.

Moreover, we think the fact, which must be within the experience of every one, that the mind may be acted upon not in *two* opposite directions only, but in *many* different ones, conclusive against this theory. Because if two brains are required for a *pro* and *con* argument in regard to any particular action, half-a-dozen brains must be wanted, when six different courses of action suggest themselves to the mind, which is urged by a distinct set of motives in reference to each.

We consider Dr. Wigan's mistake, for such we cannot but regard it, with respect to the possible existence of two volitions in the mind *at the same moment,* as a most fundamental one; vitiating his whole train of reasoning. That different and opposing volitions may succeed each other with extraordinary rapidity, is a fact perfectly clear; but that two *volitions* are ever in the mind at one period, and carry on a contest there, appears to us totally irreconcileable, not only with the experience of every one, (when carefully analysed,) but also with the idea of single individuality. We believe that in all instances, in which two volitions appear to be acting at once upon the body, or in which a series of apparently voluntary actions is taking place when the mind is occupied upon a different subject,—it is capable of proof that one of the actions is referrible to the automatic group already indicated, or that it has become so habitual, as to be performed through the same channel. This we take to be the case, for example, in regard to the performance of a piece of music whilst a conversation is being maintained; or the act of reading aloud, when the mind is wholly bent upon some train of thoughts of its own.

Dr. Wigan may perhaps say, that we differ in regard to the use of the term *volition;* and that what *we* have described as powerful or irresistible *motives,* are what *he* means by *volitions.* We shall therefore attempt to explain our meaning more clearly; and this we can do more satisfactorily by a simile than by a definition. Let us suppose a mass of matter acted upon by two forces, A and B, which pull it in contrary directions; the mass will remain undisturbed, if these forces be equal; but if either predominate, it will move towards the side on which the traction is the strongest. The *forces* are, in our apprehension, the *motives;* the *resulting movement* represents the action of the *will.* Now there may be any number of distinct forces, tending to draw the mass in different directions; and these, too, may balance one another so exactly, as to produce no movement; but, if one set predominate, the mass will be drawn towards them, in a direction which is called the *resultant,* being found by a process which takes into account the combined amounts and directions of all the different forces. Just so is it in regard to the operations of the mind; for however diversified the motives, and however numerous the modes of action towards which they severally draw, the volition which results from them is as *single* in its nature as the resultant in mechanics, and is, like it, the result of the combined operation of all the forces in action. The great difficulty in forming correct judgments in complex cases, is *not* in striking the balance between the different motives, when their respective cogency has been determined, but in assigning to each its determinate weight, and in taking into the account everything that ought to have a bearing upon the decision,—a thing not by any means so easy of accomplishment in morals as in physics.

We trust that we need not urge anything further upon this point; but that we have succeeded in communicating to our readers the chief grounds of our belief, that none of the *ordinary* mental phenomena require the hypothesis of "two distinct brains," and that many of them are inconsistent with it. We shall now pass on to consider the application of Dr. Wigan's hypothesis to these abnormal phenomena of mind, which he regards as peculiarly elucidated by it; stopping for a few moments, however, to notice a case, which seems to be given by Dr. Wigan as one of "double volition;" although we must confess ourselves at a loss to discover in what part of it any such double volition is indicated. This case (pp. 58 to 64) is too long for quotation, and we can only indicate the heads of it. We much doubt whether it be not the relation of the worthy Doctor's own experience; so much does the form of narration (avowedly furnished by the patient) bear the impress of Dr. Wigan's peculiar style; and such a devout believer does the "intimate friend" declare himself to be in the duality. He describes himself as having always been morbidly sensible to misconstruction; and as having been placed in situations which gave the appearance of guilt, or, at any rate, of culpable indifference to opinion. When alone and in depressed spirits from diffused gout and dyspepsia, he would sink into a reverie about some imaginary crime; and would plan the means of defending himself. "The whole of this time I knew perfectly and continuously that it was an entire delusion; . . . . yet, on going into agreeable and elevated society, and taking two or three glasses of wine, I generally became not merely cheerful, but almost hilarious." On one occasion, "a sudden combination of unfortunate events having produced great aggravation of my habitual distress from moral and physical causes, I felt myself entirely unable to control the expression of my mental agony in the presence of persons from whom it was of the greatest importance to conceal it." Walking down to the sea-shore, his mind in a perturbed and excited state, and his eye catching the long stream of silvery light on the water, reflected from the moon, he said to himself almost unconsciously, "that shining path is the road to happines, I will follow it."

Seeing a boat on the beach, only a little way above the water, he felt impelled to launch it, and to follow this direction. A conviction of the absurdity of this proceeding, and of the delusive nature of the impulse, on the other hand, tended to restrain him, and at last, finding no other way of checking himself, he threw himself on the ground, and kicked his heels in the air, ("my feet," he says, "continued to imitate the action of walking,") until, after the lapse of an hour, he became tolerably well cooled down, and was able to get up and walk home, without again approaching the seducing boat. Now this case appears to us perfectly simple. The disordered condition of certain feelings, resulting from the combined action of external causes and bodily ailment, caused the mere sight of an object to suggest an action, and even to urge towards it, which his better feelings and reasoning powers told him was absurd. The two sets of motives had a fierce conflict for the mastery; his volition first yielded to one, and was then overcome by the other; and at last, like the barber, he sought safety by voluntarily interposing a difficulty sufficient to prevent the accomplishment of the impulse.—We see no difference between the case here alluded to, and the very frequent one of temptation to suicide, as a consequence of mental depression. The propensity may result from very trifling causes, acting on a mind depressed through bodily disorder; or it may be the consequence of severe mental distress, arising entirely from external causes; or it may be simply the result of the imitative tendency; in short, it may arise from very numerous motives; and numerous motives will present themselves of an antagonistic character. The relative strength of these will decide the *will;* which may be swayed to one side or the other, according to the vividness of particular impressions. Not unfrequently, the resistance long kept up by the better feelings will be overcome, by the occurrence of a tempting opportunity; the temptation thus added makes the urgency so great, that the antagonism is overcome, the fixed determination is made to execute the act, and the will performs the requisite movements. Now here, we can see no proof or indication of a double volition. The contest between the two opposite lines of conduct may have been long raging; but it is really a contest of numerous diversified *motives;* and a precisely similar struggle may take place, as we have already remarked, in cases where the choice is not restricted between *two* things, but where *several* different courses present themselves for the decision of the individual. If Dr. Wigan had borne this fact in mind, we think that he could not have come to the conclusions which he has expressed.

The following are the propositions in which Dr. Wigan develops the application of his views to the better understanding and treatment of insanity.

10. "(5.) That when one of the cerebra becomes the subject of functional disorder, or of positive change of structure, of such a kind as to vitiate mind or induce insanity, the healthy organ can still, up to a certain point, control the morbid volitions of its fellow.

11. "(6.) That this point depends partly on the extent of the disease or disorder, and partly on the degree of cultivation of the general brain in the art of self-government.

12. "(7.) That when the disease or disorder of one cerebrum becomes sufficiently aggravated to defy the control of the other, the case is then one of the commonest forms of mental derangement or insanity; and that a lesser degree of discrepancy between the functions of the two cerebra constitutes the state of conscious delusion.

13. "(8.) That in the insane, it is almost always possible to trace the intermixture of two synchronous trains of thought, and that it is the irregularly alternate utterance of portions of these two trains of thought, which constitutes incoherence. That of two distinct simultaneous trains of thought, one may be rational and the other irrational, or both may be irrational; but that in either case the effect is the same, to deprive the discourse of coherence or congruity. Even in furious mania, this double process may be generally perceived; often it takes the form of a colloquy between the diseased mind and the healthy one, and sometimes even resembles the steady continuous argument or narrative of a sane man, more or less frequently interrupted by a mad man; but persevering with a tenacity of purpose in the endeavour to overthrow the intruder.

14. "(9.) That when both cerebra are the subjects of disease, which is not of remittent periodicity, there are no lucid intervals, no attempt at self-control, and no means of promoting the cure; and that a spontaneous cure is rarely to be expected in such cases.

15. "(10.) That, however, where such mental derangement depends on inflammation, fever, gout, impoverished or diseased blood, or manifest bodily disease, it may often be cured by curing the malady which gave rise to it.

16. "(11.) That in cases of insanity not depending on structural injury, in which the patients retain the partial use of reason (from one of the cerebra remaining healthy or only slightly affected,) the only mode in which the medical art can promote the cure beyond the means alluded to, is by presenting motives of encouragement to the sound brain to exercise and strengthen its control over the unsound brain." (pp. 26-28.)

That the phenomena of insanity, were they exactly as Dr. Wigan represents them, would be easily and on the whole satisfactorily explained by the "Duality" theory, and would afford support to it, must be apparent to the reader of the foregoing quotation; in which a very ingenious case is presented for our consideration. And Dr. Wigan certainly displays considerable art in the manner in which, having *in limine* seduced his reader into a provisional belief, he gradually fences him in by accumulating arguments, until he finds it difficult to extricate himself. We may even confess that, having been ourselves desirous to do him full justice, we yielded ourselves in the first instance to his enticements; and that we closed the book after a first perusal with the feeling that he had made out a probable case. But being determined to do justice to ourselves and our readers, as well as to our author, we recommenced our examination more critically; and the result has been very different. Dr. Wigan's mode of argument seems to us like that of a besieging general, desirous of taking a fortress, who should say to its commander,—"We cannot try our strength fairly, whilst you remain shut up in your castle; do me the favour to put me in possession of it, and to take my place, and then see whether *I* can hold out against *you*."

The point which struck us, on our first examination, as most in Dr. Wigan's favour, is the very frequent existence of an insane propensity or delusion, in conjunction with a conviction of the morbid nature of the feeling, which gives to the individual the power of *acting* sanely. The idea that, in such cases, the disordered condition is confined to one cerebral hemisphere, and that the other, being still unaffected, controls its impulses, —has, to say the least, a strong *appearance* of probability. But we shall try to show that it is no more than an appearance, and that it will not stand examination. Let us take, as the simplest case, the existence of some exaggerated *propensity*, which prompts to a certain course of action; the individual is aware of the disorder, restrains himself from yielding to the propensity, keeps out of the way of temptation, and endeavours to conceal his state from observation. As the *acquisitive* propensity has recently been brought rather strongly before the public, we may take this as an example. Now the individual troubled with this unfortunate propensity may be in other respects a very moral benevolent person, who would shudder at the thought of wilfully violating the Divine law or of injuring man, and would not less dread the shame of exposure before his fellow-creatures; and yet the propensity may be so strong, as to be almost uncontrollable by these or any other motives, giving rise to the most harassing conflicts and the deepest anguish. Now in such a state, what is it that arms the will to resist the imperious mandates of the exaggerated propensity? Is it the *moderate* acquisitiveness of *one* side, keeping down the *excessive* acquisitiveness of the other? Surely not. It is the force of other propensities, and of reason, which are set up as antagonists; and which, if strong enough, keep down by their united power the struggles of the insane tendency. The human mind has the power of strengthening certain tendencies, by frequently bringing them into use, and of weakening the force of others, by compelling them to inaction; just as it can alter the configuration of the body, strengthening some parts and weakening others, according as it brings the muscles of the former into frequent and powerful action, and keeps those of the other in repose. This is the state in which man's power over himself in preventing insanity, is most strongly exhibited. But the hypothesis of the two cerebra does not help us in the least to explain it. If the control of the disordered propensity be not exercised by the sound propensity on the other side, but by the antagonism of *another* set of motives,—and all experience shows that this last is the case,—then it may be just as safely affirmed that the front of the brain is acting against the back, or the vertex against the base.

Dr. Wigan seems to exclude from his category the instances in which the animal passions prompt to a certain course of action, from which they are restrained by the will; but we cannot see in what the essential difference lies, between the excessive development of the sexual propensity, for example, and the exaggeration of any other tendency. This may become so strong as to be ungovernable by any restraint, save physical coercion: but suppose it to stop short of this, and to be restrained by the action of moral considerations upon the will;—surely in such a case there is no reason whatever to believe, that one half of the encephalon is disordered, and that the sound side perceives the morbid state of the other. Then why should this supposition be entertained regarding other propensities and feelings, less directly *animal* in their tendency, but precisely analogous to them in the mode in which they operate on the intellect?

Now we are well convinced that the progress of our knowledge in regard to insanity, will in time enable us to see, that nearly, if not quite, all the cases generally set down as monomania, really consist, in the first instance at least, of disorder of one or more of the *moral* feelings, tendencies, or propensities; and that the *intellectual delusion* which is frequently the prominent symptom of such affections, is the consequence of this. We believe that, to this conclusion, those who have the best opportunities of observation, are gradually tending; and that it must ultimately have an important influence, both on our therapeutic measures, and on our views of the ordinary or normal operations of the mind. The progress to such states, from those which we have just been describing, is perfectly simple. The insane propensity gains strength, whether from the want of self-control and the habit of yielding to its promptings, or from the progress of the physical change to which it was due in the first instance; the will can no longer be kept under the control of the opposing feelings, and yields itself up unresistingly to the domination of the tyrant; and all the actions exhibit its influence. Now the trains of thought, which occupy the mind in the intervals of action, are no less under the dominion of this morbid power, than the will itself. We possess in health (and nearly all metaphysicians are agreed that this is a distinguishing feature of the *human* mind,) a considerable power over the sequence of our thoughts; and so long as the mind can view things as they really are, if it be only at intervals, it can correct the representations of them which are made through the distorted medium of its disordered feeling. But when this feeling gains so much comparative strength,—whether by the weakening of the rest of the mental fabric, or by a positive increase in itself,—as to take full possession of the thoughts, it represents every thing that takes place around in a wrong light, and furnishes wrong data to the reasoning processes; and even the memory and experience of the past are prevented from furnishing the correction they might be expected to afford, since they too are viewed through the same distorted medium.

In the comprehension of such cases, we cannot see that the hypothetical "duality" gives us the least assistance. If we may localize the passions, propensities, and moral feelings of all, as the phrenologists tell us,—or even if we only go so far as to admit that there is such a localization, without admitting that it has yet been satisfactorily made out,—the action of the brain in this train of occurrences may be explained just as well (or we should say much better,) on the hypothesis that both the corresponding organs are affected together, but are in the first place antagonized by others,—as on the idea that the right and left brains are quarrelling for mastery. And *our* idea would further derive confirmation from the fact,—which opposes Dr. Wigan's in the same degree,—that mental conditions of the nature we have been describing are very frequently connected with a disordered state of the system in general, which will act upon the brain through the blood. Now such affections, whether resulting from the retention of excretions, or from the introduction of new morbid elements into the blood, must be admitted to be most probably *symmetrical* in their action as respects the brain; and we cannot admit that they are almost uniformly otherwise, without strong proof. Let Dr. Wigan observe the gradual access of the state of intoxication, as glass after glass of wine mounts (to use a phrase which is probably as accurate in a physical sense as it is in its common meaning,) into the brain. At the beginning of the process, the *patient* feels nothing but a pleasing excitement; his ideas flow more freely and strongly than usual, and he possesses an unwonted command of language, which enables him to express them with fluency. He is perfectly aware of his situation, and rejoices in it; and either aims at keeping up the same pleasing excitement by continued libations; or, if his higher moral feelings predominate, he subdues his sensual inclination by their aid, and indulges no longer. According to Dr. Wigan, he is, at this stage *drunk* on one side of his head, and *sober* on the other. If he proceed, his ideas become altogether confused, his expressions incoherent, he loses his control over the current of his thoughts, and he becomes for the time insane; not, however, having his mind possessed by any fixed delusion, because his madness results from a disturbance of all his faculties, sensory, moral, and intellectual, rather than of any one. He then becomes drunk on *both* sides. Now this we hold to be a most legitimate illustration of Dr. Wigan's theory; and cannot charge ourselves with the least violence in so applying it. Many of our readers, we think, will consider its unsoundness thus proved, as by a *reductio ad absurdum;* but if Dr. Wigan and his partisans affirm that the representation we have given is a correct one, and that a man in a state of incipient intoxication *is* drunk on one side and sober on the other, we then ask for the *proof* of that, which all analogy opposes; namely, the restriction of the *alcohol* introduced into the blood, to *one* side of the brain, though even for a short time only.

We may sum up our argument as follows: instead of being required by the phenomena of conscious delusion, and the mental operations connected with it, to believe in the duality of the mind, and the complete independence of the two sides of the brain, we think them even more satisfactorily explicable on the idea, that the mind and the brain alike consist of one whole; and that where an opposition exists between the sane and the insane tendencies, it is *not* between the two tendencies or faculties of the *same* kind allocated in opposite sides of the brain, but between the morbidly-exaggerated tendencies on the one hand, and those of an *opposite* character, which should keep them under control, and which may be allocated on the same side of the brain.

There is one more class of phenomena, on which Dr. Wigan lays considerable stress; but which we do not consider that his theory at all helps to explain; that, namely, of spectral illusions. These are delusions of *sense,* in the same manner as those to which we have already alluded are delusions of *intellect.* So long as the mind has power, by dwelling on its past experience, to prove to itself the fallacy of their indications, so long they may pass and repass without disordering its other processes. But let it be weakened from fatigue or depression, so that the comparison cannot be efficiently made; and it yields. We perceive no more reason for supposing that one cerebrum, in such cases, *sees* the objects, and that the other reveals the fallacy of the appearances, than for supposing that, when Dr. Wigan goes to see a conjurer, who induces him to place a bank-note in his hands and then apparently burns it, one of the worthy Doctor's brains witnesses the combustion, whilst the other tells him that the conjurer knows better than thus to sacrifice his property. The only difference is that, in the one case, the cause of the illusion is an external sleight of hand, whilst in the other it is some change within the individual. In every other respect, the case seems to us the same.

We need not dwell upon the conflicting tendencies, so frequently and strikingly manifested in insanity, which are adduced by Dr. Wigan as main supports of his theory; for they are only those of the sane mind in an exaggerated form; and all that has been said in reference to the latter is, therefore, equally applicable to them.

Perhaps the most important kind of evidence in favour of Dr. Wigan's views, is that which is afforded by the phenomena of sleep-waking, and of double consciousness;—or as Dr. Wigan prefers to call it alternate consciousness, "the person being in a manner two individuals, as far as sensation and sense of personal identity are concerned." Some forms of these remarkable conditions, we noticed at some length in our last Number. The most curious, perhaps, are those in which the two states are so distinct, that not a thought or memory of the one passes into the other. The following classification of *analogous* phenomena, formed by Dr. Wigan for the better explanation of his views, expresses, we believe, the principal forms of those disordered states of mind, which throw most light upon these peculiar conditions.

"A. We know by innumerable examples, that a sudden physical shock, or a blow on the head, shall reduce the healthy and acute brain of a profound scholar to a state wherein he has all the mental characteristics of childhood—is pleased or offended by trifles, so apparently insignificant that in his previous state they would not excite the most evanescent attention—his sensations and perceptions are still perfect, but his reasoning powers are gone.

"B. In other cases a similar accident shall obliterate portions, or the whole, of his acquired knowledge—he will lose, for example, one language and retain others, or he may lose all; and on his recovery from the physical effects of the accident, he has to begin life again, and proceed to acquire information in the same mode as a child, though with a much slower progression.

"C. These effects arise sometimes also from a moral shock, such as the sudden communication of afflicting news—terror—detection in crime, or any other analogous cause, equally or indifferently whether the cause be moral or physical; the brain is entirely spoiled, temporarily deranged, only slightly injured; or in gradation from one to the other, losing one or more of its functions, and one or more portions of acquired knowledge.

"D. After such effects have lasted a considerable time, and have or have not been accompanied or followed by any of the usual forms of mental aberration, or of imbecility, the whole powers of the brain may be restored either gradually or in an instant—the watch may resume its motion." (p. 395.)

It is only necessary, according to Dr. Wigan, to explain these facts according to the Duality-theory, to get at the whole rationale of alternate consciousness; which he believes to be comprised in the following propositions:

"1. If then my doctrine of the entire completeness and sufficiency of each brain as an instrument of mind be firmly established, it follows, so plausibly as to be almost certain, that any of the states A, B, C, and D, and many intermediate modifications of them, may spontaneously occur in one brain, leaving the other entirely unaffected. We see an example of this in hemiplegia, or paralysis on one side only.

"2. One brain may be subjected to one of these changes, and the other brain may have its powers and functions changed or modified in a different manner.

"3. One brain may be reduced to the state of childhood (state A,) and the other remain in its ordinary state.

"4. One brain may be in the state A or B, and the other may have its functions suspended or modified by a greater or less degree of torpor, as in sleep, catalepsy, extasis, &c.

"5. One brain may be in the state of childhood, and the other torpid and unconscious." (p. 396.)

There is certainly much in this mode of explaining the phenomena of Double or Alternate Consciousness, that, if we admit the possibility of connecting *mental* and *physical* conditions to this extent, seems highly probable. And it is certainly an argument in favour of the view in question, that we never hear more than *two* such conditions as presented by the same individual. It will be observed that in cases of complete alternate consciousness, what we stated at the outset as the only notion of the duality of the mind—the existence of two *egos* or consciousnesses in the same body—is to a certain extent fulfilled. We say to a certain extent, however, because these two states are *never coexistent;* and until two *egos* could be shown to be in action *at* the same time, we can see no cause (perhaps it would not even *then* exist) for unhesitatingly allocating them in the two sides of the cerebrum. We know too little of the connexion between mind and body, or of the effects produced upon the latter by disease, to be able to assign the real causes of *any* mental disturbance produced by disease or accident. For example, a man falls upon his head—on one side it may be,—and henceforth loses the memory of a particular language. Now it is not at all easy to understand how the corresponding part of *each* hemisphere should be affected in the same manner, by a blow on *one.* If there be any localization of such a memory at all, such *must* be supposed to occur; unless we imagine that the effect is exerted upon that *central* organ which we believe to be the peculiar residence of the mental essence—if such essence exist,—or in other words, the peculiar seat or centre of mental phenomena.

Our object has been, throughout, to prove that Dr. Wigan has not made out his case; not to demonstrate that he is wrong. We regard the question as furnishing a very good *peg* on which to hang observations; and in that form it was put forward by Dr. Holland, in his 'Essay on the Brain as a Double Organ.' We have not said a word respecting the question of Dr. Wigan's originality; because he over and over again affirms this to be a point on which he is perfectly careless. But we think it right to state that the question does not seem to be advanced one step by Dr. Wigan's publication. His dogmatism on questions, as to which no certainty *can* be attained, goes for positively nothing; and we should ourselves rather follow Dr. Holland as our guide in the inquiry. It is but just to Dr. Wigan to state, that he fully recognizes the value of Dr. Holland's suggestions, as far as they go: and dedicates his volume to him as a tribute of respect to "a profound thinker and able physician, a scholar, a philosopher, and a gentleman." He also mentions Mr. Barlow's little treatise "On Man's Power over himself to prevent or control Insanity," (noticed by us vol. XVII, p. 245,) as having been the immediate cause of the development of his long-cherished views in their present form. In fact he seems to have been restrained by the fear of being charged with materialism, atheism, &c., &c.; and to have been encouraged by the clerical example to make known his speculations. We hope that the time is going by, when this cry will be raised against *any* fearless enunciation of what he believes truth. The inductions of science are not to be tested by their conformity with our preconceived notions, as to the nature of the divine government, or the connexions of matter and spirit; but by the degree in which they are supported by those *facts,* which are a revelation of the Deity's mind and will, no less than his written Word. It is by these, and these alone, that we have tried Dr. Wigan.

